# The role of spatial self-organization in the design of agroforestry systems

**DOI:** 10.1371/journal.pone.0236325

**Published:** 2020-07-21

**Authors:** Omer Tzuk, Hannes Uecker, Ehud Meron

**Affiliations:** 1 Department of Physics, Ben-Gurion University of the Negev, Beer Sheva, Israel; 2 Institut für Mathematik, Universität Oldenburg, Oldenburg, Germany; 3 Department of Solar Energy and Environmental Physics, Blaustein Institutes for Desert Research, Ben-Gurion University of the Negev, Beersheba, Israel; Vrije Universiteit Amsterdam, UNITED KINGDOM

## Abstract

The development of sustainable agricultural systems in drylands is currently a crucial issue in the context of mitigating the outcomes of population growth under the conditions of climatic changes. The need to meet the growing demand for food, fodder, and fuel, together with the hazards due to climate change, requires cross-disciplinary studies of ways to increase livelihood while minimizing the impact on the environment. Practices of agroforestry systems, in which herbaceous species are intercropped between rows of woody species plantations, have been shown to mitigate several of the predicaments of climatic changes. Focusing on agroforestry in drylands, we address the question of how we can improve the performance of agroforestry systems in those areas. As vegetation in drylands tends to self-organize in various patterns, it seems essential to explore the various patterns that agroforestry systems tend to form and their impact on the performance of these systems in terms of biomass production, resilience to droughts, and water use efficiency. We use a two-soil-layers vegetation model to study the relationship between deep-rooted woody vegetation and shallow herbaceous vegetation, and explore how self-organization in different spatial patterns influences the performance of agroforestry systems. We focus on three generic classes of patterns, spots, gaps, and stripes, assess these patterns using common metrics for agroforestry systems, and examine their resilience to droughts. We show that in contrast to the widespread practice of planting the woody and herbaceous species in alternating rows, that is, in a stripe pattern, planting the woody species in hexagonal spot patterns may increase the system’s resilience to droughts. Furthermore, hexagonal spot patterns reduce the suppression of herbs growth by the woody vegetation, therefore maintaining higher crop yields. We conclude by discussing some limitations of this study and their significance.

## Introduction

**Fig 1:**
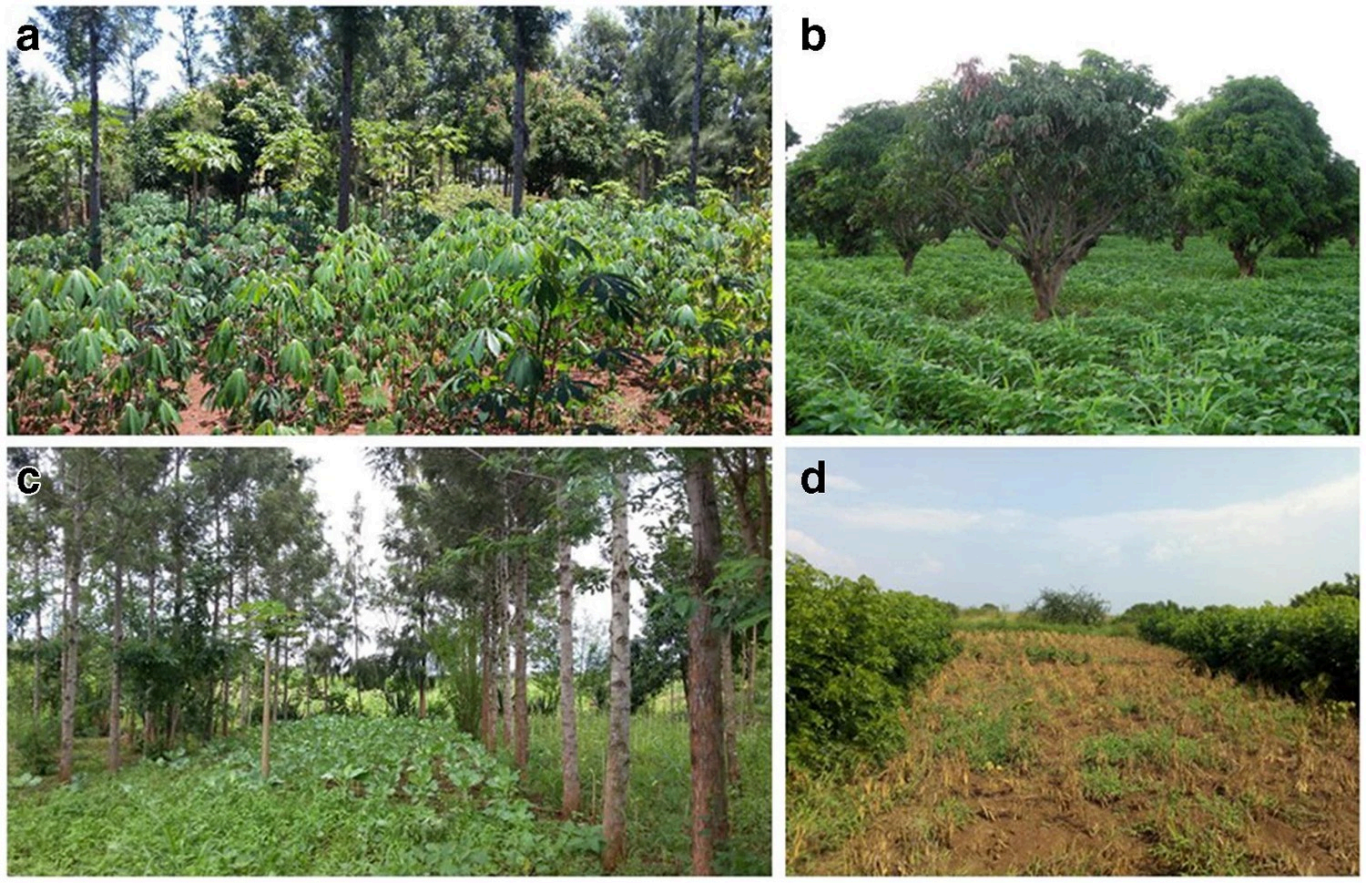
Illustrative examples of agroforestry practices commonly used in sub-Saharan Africa. From [9].

Climatic changes worldwide call for exploration of innovative designs for agriculture and ecosystem management. Currently, about 40% of Earth’s land is classified as drylands, where precipitation is on a critical balance with evapotranspiration [1]. These regions are estimated to support more than 38% of the world’s population. Severe land degradation of 10–20% of drylands [2] would affect a large part of the population in these areas. Furthermore, according to projections, drylands are in accelerated expansion and will cover half of the global land area by the end of the 21^st^ century [3]. The risk of further degradation and expansion of drylands, along with the forecasts of population growth in those areas, calls for developing methods of sustainable use of resources. Agroforestry is a land-use system in which woody species (e.g., trees and shrubs) are grown in a spatial configuration together with crops or pastureland. A few examples are shown in Fig 1. These systems have been suggested as sustainable methods for agricultural and forest production, that may mitigate the adverse impact of climate change on agriculture [4]. Specifically, dryland agroforestry has been demonstrated as an advantageous method over conventional agricultural systems in terms of soil stabilization, biodiversity, bioproductivity, and restoration of degraded lands [5–8].

There is accumulating evidence for the success of agroforestry practices in arid and semi-arid areas throughout the world. For instance, growing nitrogen-fixing trees with crops was proved to increase crop yields [10], and combining agroforestry practices with rain-harvesting techniques increases green water-use efficiency (the proportion of rainfall used for plant transpiration) [11, 12]. However, given the increasing environmental stress due to climate change in arid and semi-arid regions, including the more frequent occurrence of climate extremes, such as prolonged droughts, it is necessary to continue improving the water-use efficiency, resilience, and persistence of agroforestry systems in those regions.

A primary objective of agroforestry systems is to mimic the favorable environmental conditions found in natural systems while maintaining a high agricultural value for farmers [13]. A central agroforestry hypothesis proposed by Cannell et al. [14] states that woody species must not exploit the resources taken by the crops in order to achieve an overall positive gain of the system. Several studies have suggested that agroforestry systems that imitate the resource use patterns of natural ecosystems, and in particular the partitioning of water resources, may reduce adverse effects of competition between the resident species [15, 16]. A widespread application of agroforestry practices is the intercropping of herbaceous vegetation, typically of staple crops, between woody–vegetation plantation [17]. Some of the desired outcomes of such woody species intercropping include: increase in the total biomass–productivity of the system (of both woody and herbaceous species) due to a more efficient exploitation of water from deep-soil layers, higher biodiversity of the system as a whole, improved soil fertility, better nutrient cycling, amelioration of microclimate, and higher resilience of the system to perturbations [18]. However, various negative feedbacks between the species may reduce the profitability of agroforestry plantations. Such adverse effects may be the result of several factors, including competition over water and excessive shading. Thus, a major aim of agroforestry research in drylands is to minimize the negative feedbacks between the woody and herbaceous species.

A salient feature of vegetation in drylands is its property of spatial self-organization, in regular patterns. Spatial self-organization in dryland vegetation may arise from positive feedback loops between local vegetation growth and water transport towards the growth location, where the latter can be overland water flow, water conduction by laterally distributes roots, and soil-water diffusion [19]. Vegetation models that capture one or more of these positive feedback loops have been successful in reproducing vegetation patterns observed in drylands [20–26]. The redistribution of water by patterned vegetation increase the resilience of the ecosystem to prolonged droughts by providing an extra source of water besides direct rainfall, namely, the water that vegetation patches draw from their bare-soil surroundings [19]. Some of these models have been extended to describe woody-herbaceous systems [27–29].

As experiments on real agroforestry systems can span many years, it is necessary to use models to facilitate system design. There is a wide array of process-based models that are employed in agroforestry system design, and capture in high detail the resources flow to the tree and crop species [30]. However, to the best of our knowledge, no agroforestry modeling scheme addresses the spatial self-organization that characterize the vegetation in drylands. In the prevalent modeling schemes for agroforestry, the spatial template of the vegetation is a fixed condition, as the assumption is that the farmers control the growth of the tree and crop species entirely. Although these models are useful for optimizing the design of tree-crop templates and species mixture on short time-scales, they do not allow the detection and characterization of long-term self-organization trends.

Accounting for spatial self-organization processes can elucidate resource use patterns that may be beneficial for improving the performance of drylands agroforestry systems. In this study, we propose a spatially explicit model of a woody–herbaceous system that aims to reveal long-term spatial self-organization of those systems. A possible avenue for the application of our study is in the design of agroforestry systems in drylands.

Drawing from the concept of biomimicry [16, 31], we suggest that agroforestry systems imitating the spatial patterns of woody–herbaceous ecosystems in semi-arid environments will be more sustainable and resilient to exogenous disturbances. By studying the relationship between deep-rooted woody vegetation and shallow herbaceous vegetation in the context of pattern formation theory, we aim to understand the alternative ways in which those systems can self-organize in space to efficiently exploit available water resources. Accordingly, our study consists of two steps: characterizing the different spatial patterns that the system would tend to converge under given environmental conditions and identifying the optimal pattern in terms of productivity, water-use efficiency and resilience to droughts.

The paper is organized as follows. We begin with a description of the methods we use in our study. These include the mathematical model we use, numerical methods of solving it, and the performance metrics we use to compare alternative pattern states. We then describe our results, which include (a) a bifurcation diagram of the stable states of the systems over a 2D domain for a range of mean annual precipitation values, (b) the comparison of the alternative states using the agroforestry performance metrics, and (c) numerical studies of the resilience of the alternative patterns to decrease in precipitation. A discussion of these results and their limitations concludes the paper.

## Methods

### A new model for woody–herbaceous dynamics

**Fig 2:**
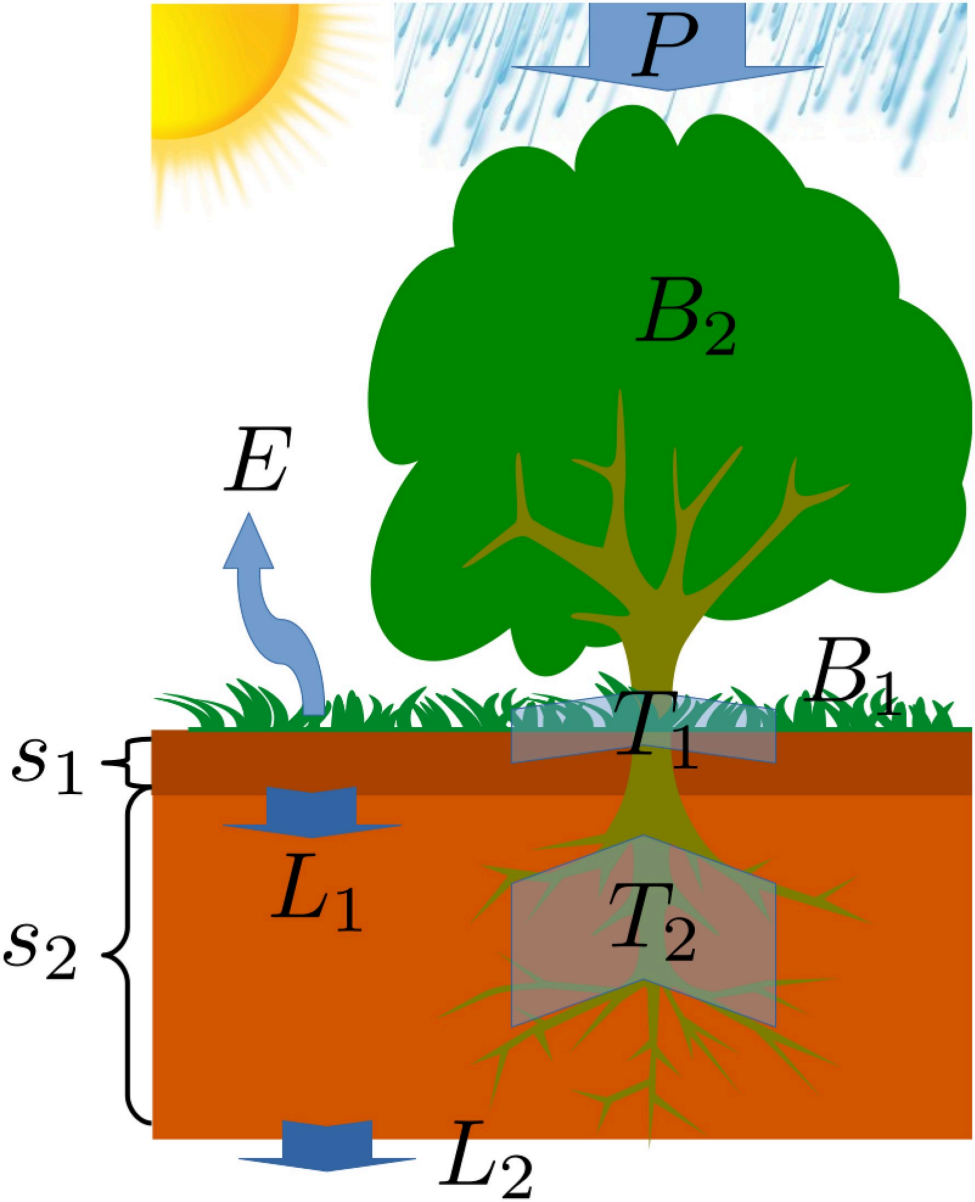
A schematic representation of the model. Along with the four dynamics variables *B*_1_,*B*_2_,*s*_1_, and *s*_2_, the model takes into account the mean annual precipitation *P*, the evaporation from the upper soil later *E*_*v*_, infiltration from upper soil layer to lower one *L*_1_, and the infiltration from the lower soil layer to deeper layers *L*_2_. The transpiration terms *T*_1_ and *T*_2_, of the herbaceous and woody species respectively, are dependent on the aerial biomass density of each species, together with the relative soil moisture in each soil layer.

Our model is an extension of the vegetation model presented by Baudena et al. [32] to two plant species. This model takes into account the depth dimension of the soil by considering two soil layers, an upper layer of depth *Z*_1_ and a lower layer of depth *Z*_2_. We consider a woody-herbaceous system [27], and denote the herbaceous biomass by *B*_1_ [*kgm*^−2^] and the woody biomass by *B*_2_[*kgm*^−2^]. We simplify the model introduced by Baudena et al. by assuming sandy soil, which is characterized by high infiltration rates. This allows to neglect overland water flow and eliminate the equation for surface water [19] In addition to the two biomass variables, the model consists of two water variables: relative soil moisture level in the upper soil layer *s*_1_, and in the lower soil layer *s*_2_. These variables correspond to the ratios of water volume to soil pore volume, and their ranges are between zero and one. We will consider an ecosystem in which there is a partial or full niche separation of the water resources between the woody and herbaceous-species. While the herbaceous-species water uptake is from the upper layer only, the woody-species water uptake can be both from the lower layer and the upper layer. A schematic representation of the model is presented in Fig 2. The model is spatially explicit and includes four partial differential equations that describe the evolution in time of the four variables of the system:

$$\begin{array}{ccc} \partial_{t}B_{1} & = & G_{B_{1}}(B_{2},s_{1})B_{1}(1-\frac{B_{1}}{K_{1}})-M_{1}B_{1}+D_{B_{1}}\nabla^{2}B_{1}, \end{array}$$ (1)

$$\begin{array}{ccc} \partial_{t}B_{2} & = & G_{B_{2}}(s_{1},s_{2})B_{2}(1-\frac{B_{2}}{K_{2}})-M_{2}B_{2}+D_{B_{2}}\nabla^{2}B_{2}, \end{array}$$ (2)

$$\begin{array}{ccc} nZ_{1}\partial_{t}s_{1} & = & P-E_{v}(B_{1},B_{2},s_{1})-L_{1}(s_{1})-T_{1}(s_{1},B_{1},B_{2})+nZ_{1}D_{s}\nabla^{2}s_{1}, \end{array}$$ (3)

$$\begin{array}{ccc} nZ_{2}\partial_{t}s_{2} & = & L_{1}(s_{1})-L_{2}(s_{2})-T_{2}(s_{2},B_{2})+nZ_{2}D_{s}\nabla^{2}s_{2}\;, \end{array}$$ (4)

where *t* is time, and ∇^2^ = ∂^2^/∂*x*^2^ + ∂^2^/∂*y*^2^.

Eqs (1) and (2) describe the dynamics of the herbaceous and woody aboveground biomass, respectively. We distinguish between the woody and the herbaceous species mainly by assuming significantly higher maximum standing biomass of the woody species (*K*_2_ > *K*_1_), and significantly higher growth and mortality rates of the herbaceous species (Λ_1_ > Λ_2_, *M*_1_ > *M*_2_) [27]. Additionally, we consider the reduction of the herbaceous-species growth rate due to shading from the woody-species by assuming the following dependence of $G_{B_{1}}$, the actual herbaceous growth rate, on the woody species biomass:

$$\begin{array}{c} G_{B_{1}}=\Lambda_{1}{\left(1+E_{1}B_{1}\right)}^{2}(1-\frac{B_{2}^{2}}{B_{2}^{2}+B_{r}^{2}})nZ_{1}s_{1}\;, \end{array}$$ (5)

where Λ_1_ is the nominal growth rate of the herbaceous species, *E*_1_ represents the augmentation of the herbaceous-species roots per unit above-ground biomass (i.e. a measure of the root-to-shoot ratio [27]), *n* is the soil porosity, and *B*_*r*_ is a reference biomass for the woody species, beyond which the effect of the reduction of sunlight exposure to the herbaceous species becomes significant. The woody-species growth rate $G_{B_{2}}$ is given by:

$$\begin{array}{c} G_{B_{2}}=\Lambda_{2}{\left(1+E_{2}B_{2}\right)}^{2}(\theta nZ_{1}s_{1}+nZ_{2}s_{2})\;, \end{array}$$ (6)

where Λ_2_ is the nominal growth rate of the woody species, and *E*_2_ represents the augmentation of the woody-species roots per unit above-ground biomass. The parameter *θ* quantifies the niche differentiation degree between the two species. The higher *θ*, the more influenced is the growth of the woody species by water availability in the upper soil layer.

Eqs (3) and (4) describe moisture dynamics in the upper and lower soil layers. *P* stands for the mean annual precipitation rate. As a first exploration of the model, we considered precipitation at a constant rate that represents the average annual rainfall. Although precipitation in these environments is usually sporadic, studies on spatially implicit models show that the difference between constant and intermittent precipitation is mainly quantitative, and it shifts the range of coexistence to lower precipitation values in respect to constant precipitation [33]. Therefore, we neglect the intermittency of precipitation to be able to improve our ability to perform a numerical investigation of the model’s behavior.

The evaporation term *E*_*v*_ takes into account the reduction of evaporation rate from the upper soil layer due to shading by the woody and herbaceous species as

$$\begin{array}{c} E_{v}(B_{1},B_{2},s_{1})=\frac{nZ_{1}s_{1}N}{1+R_{1}B_{1}/K_{1}+R_{2}B_{2}/K_{2}}, \end{array}$$ (7)

where *N* represents the maximum evaporation rate and *R*_*i*_ quantify the reduced evaporation due to shading. We assume that on bare soil the evaporation rate increases linearly with *s*_1_. The quantities *L*_1_ and *L*_2_ represent the leakage from the upper soil layer to the lower soil layer, and from the lower soil layer to deeper layers which are unreachable for the plants’ roots system. The functional form for the leakage terms is taken as [34]

$$\begin{array}{c} L_{i}(s_{i})=K_{s}s_{i}^{c}, \end{array}$$ (8)

where *c* is a constant parameter related to the pore size distribution. The terms *T*_1_ and *T*_2_ are the herbaceous and woody transpiration rates from the upper and lower layer, respectively. We take their functional forms as

$$\begin{array}{ccc} T_{1} & = & \Gamma_{1}B_{1}nZ_{1}s_{1}+\theta \Gamma_{2}B_{2}{\left(1+E_{2}B_{2}\right)}^{2}nZ_{1}s_{1}, \end{array}$$ (9)

$$\begin{array}{ccc} T_{2} & = & \Gamma_{2}B_{2}{\left(1+E_{2}B_{2}\right)}^{2}nZ_{2}s_{2}\;, \end{array}$$ (10)

where the niche separation parameter *θ* captures the degree that the woody species consume water from the upper soil layer. The functional forms of *T*_2_ and $G_{B_{2}}$, along with the diffusion term in Eq (4), establish a positive feedback loop between vegetation growth and water transport towards the growing vegetation. This feedback loop is responsible for the emergence of spatial patterns through a Turing instability [35], as further explained in the next subsection. We refer the reader to Table 1 for additional information on the model’s parameters, including their units and numerical values. Parameter values were motivated by Refs. [25, 32, 36], and are intended to describe generic woody-herbaceous systems in drylands, rather than specific realizations of such systems.

**Table 1:** Model’s parameters and their values.

Symbol	Meaning	Units	Value
*Z*_1_	Depth of the upper soil layer	*mm*	80
*Z*_2_	Depth of the lower soil layer	*mm*	420
*n*	Soil porosity	−	0.55
*K*_*s*_	Saturated hydraulic conductivity	*mmy*^−1^	7600
*c*	Leakage exponent	−	2
*N*	Evaporation rate from bare soil	*y*^−1^	4.35
Λ_1_	Herbaceous species maximum biomass growth rate per water unit	*y*^−1^ *mm*^−1^	3.03
Λ_2_	Woody species maximum biomass growth rate per water unit	*y*^−1^ *mm*^−1^	0.07
*K*_1_	Herbaceous species maximum standing biomass density	*kg*/*m*^2^	0.5
*K*_2_	Woody species maximum standing biomass density	*kg*/*m*^2^	2.0
*M*_1_	Herbaceous species mortality rate	*y*^−1^	20.0
*M*_2_	Woody species mortality rate	*y*^−1^	5.0
Γ_1_	Herbaceous species soil water consumption rate per unit biomass density	*kg*/(*y*^−1^ *m*^2^)	20.0
Γ_2_	Woody species soil water consumption rate per unit biomass density	*kg*/(*y*^−1^ *m*^2^)	5.0
$D_{B_{1}}$	Herbaceous species seed dispersal coefficient	*m*^2^/*y*	1.2
$D_{B_{2}}$	Woody species seed dispersal coefficient	*m*^2^/*y*	0.3
*D*_*s*_	Soil moisture diffusivity	*m*^2^/*y*	150.0
*E*_1_	Herbaceous species root augmentation per unit biomass	(*kg*/*m*^2^)^−1^	0.0
*E*_2_	Woody species root augmentation per unit biomass	(*kg*/*m*^2^)^−1^	2.0
*R*_1_	Herbaceous species evaporation reduction due to shading	−	0.5
*R*_2_	Woody species evaporation reduction due to shading	−	2.0
*θ*	Niche separation parameter	−	0 − 1
*B*_*r*_	Reference biomass for radiation reduction	−	1.0
*P*	Precipitation rate	*mmy*^−1^	0 − 900

### Emergence of patterns and pattern transitions along the rainfall gradient

**Fig 3:**
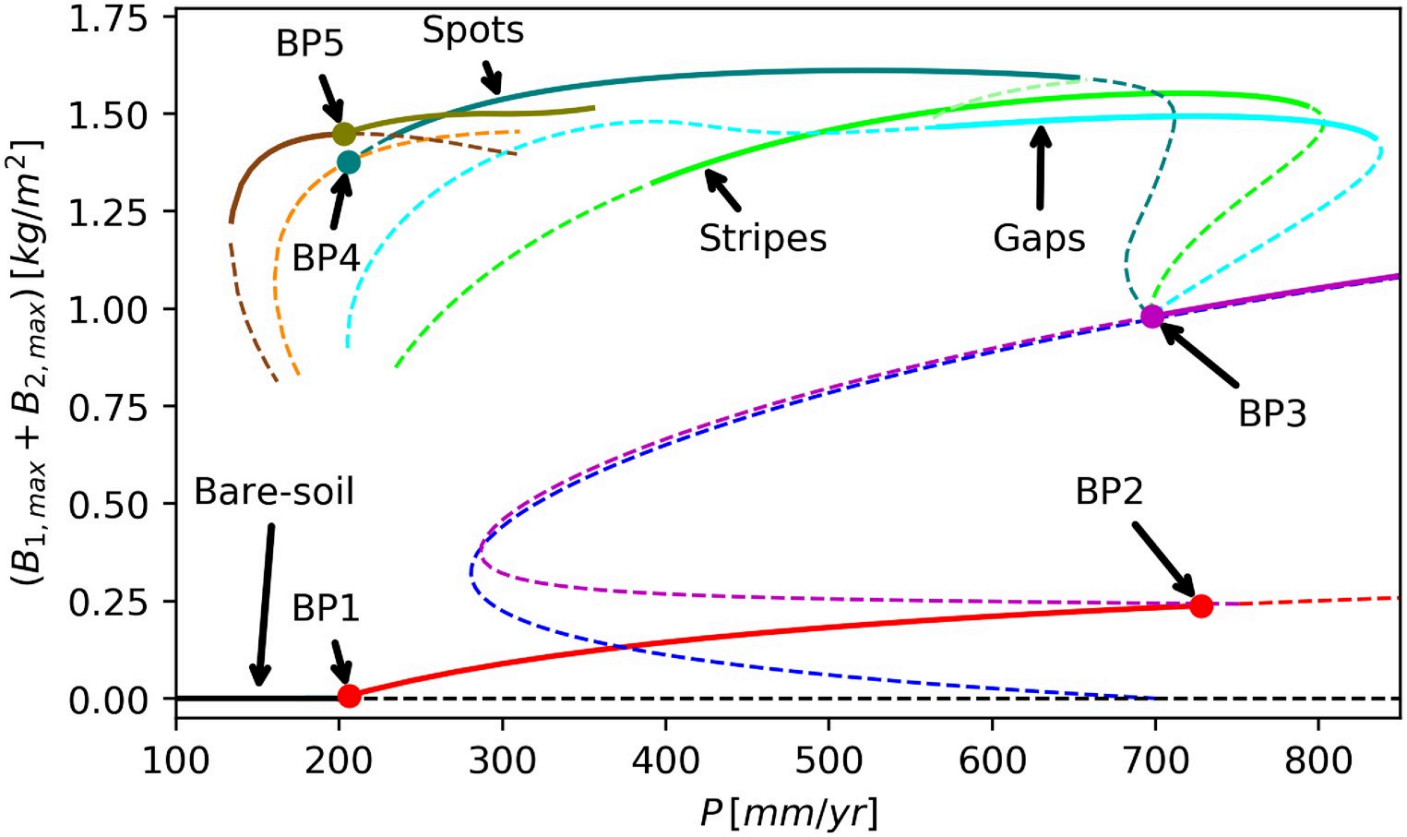
Bifurcation diagram for the model. Partial bifurcation diagram over the 2D domain Ω = (3.00 × 1.75)*m*. Stable (unstable) solutions are denoted by solid (dashed) curves. The bifurcation point BP1 marks the appearance of a solution branch describing homogeneous herbaceous vegetation(red curve) from the bare-soil solution (black curve). This herbaceous-only branch loses its stability in a subcritical bifurcation to a homogeneous mixed woody–herbaceous branch (magenta curve) at point BP2. At BP3 three patterned mixed woody-herbaceous solution branches bifurcate from the homogeneous mixed woody–herbaceous branch: gap patterns (cyan curve), stripes (green curve), and spots (turquoise curve). At BP4 the mixed-spot solution branch bifurcates to a woody-only spot branch (orange curve). An additional mixed-spot solution branch of longer wavelength (olive curve) bifurcates at PB5 to a stable woody-only long-wavelength spot branch (brown curve). A uniform woody-vegetation bifurcates from bare soil at a relatively high precipitation value (blue curve) and remains unstable throughout the range we explored.

The model described in the previous section captures a positive feedback loop between local growth of the woody species and soil-water diffusion towards the growth location [19, 37]. Incidental denser vegetation in a given location leads to stronger water uptake, which depletes the local soil-water content relative to the sparser vegetation around, and creates soil-water gradients. These gradients, in turn, induce soil-water diffusion towards the denser vegetation, a process that helps the denser vegetation grow yet denser, and makes the vegetation around yet sparser. When the water uptake is strong enough (*E*_2_ sufficiently large relative to $K_{2}^{-1}$) and soil-water diffusion is fast enough (*D*_*s*_ sufficiently large relative to $D_{B_{2}}$), this positive feedback loop can induce a Turing instability of uniform vegetation to periodic vegetation patterns. For given values of *E*_2_ *K*_2_ and *D*_*s*_/*D*_*B*_ the instability can be induced by decreasing the precipitation rate *P*. Vegetation pattern formation can then be regarded as a population-level mechanism to tolerate water stress, as vegetation patches in periodic patterns benefit from an additional water resource—the water they draw from their vicinities, be they bare soil or herbaceous-vegetation areas, where water is exploited from the top soil only. This mechanism also explains the different patterned states along the rainfall gradient (see Fig 3 and the Results section). As precipitation decreases the additional water contribution to woody patches from their bare-soil or herbaceous-vegetation surroundings, should increase in order for the woody vegetation to remain viable. That increase is achieved by means of morphological transitions, first from gap patterns to stripe patterns and then from stripe patterns to spot patterns, as these transitions increase the bare-soil areas or the areas covered by herbaceous vegetation [37]. Unlike the woody species, we assume in this study that the herbaceous species does not form patterns and therefore consider sufficiently small *E*_1_ values, specifically *E*_1_ = 0 (see Table 1).

### Agroforestry metrics

In order to compare the different states featured by the agroforestry system, we need to quantify the performance of the system in respect to chosen metrics. We chose to study two indexes that are commonly used in the context of agroforestry systems, where the woody species mainly acts as a stabilizing agent, preventing soil erosion and land degradation, rather than a second crop that contributes to the overall system’s productivity [13]:

1. *I* index: Generally, the crop is the species that the farmer is interested in maximizing its yield. The index *I* measures the advantage, or disadvantage, of growing the crop species *B*_1_ in agroforestry in comparison to growing it alone, that is, in monoculture. The index *I* is defined by the formula:
  $$\begin{array}{c} I=\frac{Y\left(B_{1,agroforestry}\right)-Y\left(B_{1,monoculture}\right)}{Y(B_{1,monoculture})}\;, \end{array}$$ (11)
  where *Y*(*B*_1,*agroforestry*_) and *Y*(*B*_1,*monoculture*_) represent crop yields, that is, the total amount of biomass produced on a given domain, in the mixed agroforestry system and in the monoculture system, respectively. The index of the monoculture system is evaluated using the stable uniform herbaceous state, while the index of the agroforestry system is evaluated using the alternative stable woody-herbaceous patterned states. The values of *I* are generally negative, as the woody species generally outcompetes the herbaceous species, thereby limiting the areas occupied by the herbaceous species. Values of *I* close to zero indicate that the agroforestry system is nearly as productive as the monoculture system. Therefore, a general objective of agroforestry system planning is to maximize *I*, such that the suppression of crop by the woody species is minimized [13].
2. *ε*_*W*_ index: This index provides a measure of the rain water-use efficiency of the agroforestry system. In general we would like to reduce the water loss caused by evaporation and deep drainage beyond the reach of the plants’ roots. To estimate *ε*_*W*_ we will calculate the rain water loss, *wl*_*i*_ for three setups, monoculture of herbaceous species (*i* = 1), monoculture of woody species (*i* = 2), and the agroforestry setup (*i* = *af*), using the expression:
  $$\begin{array}{c} wl_{i}=E_{v,i}+L_{2,i}\;. \end{array}$$ (12)
  According to this formulation, the *wl*_*i*_ measures the loss from the systems as a sum of the evaporation loss and infiltration of water to deep-soil layers that are unreachable to the woody species’ root system. In terms of *wl*_*i*_ the index *ε*_*W*_ is given by
  $$\begin{array}{c} \varepsilon_{W}=\left\langle \frac{2wl_{af}}{wl_{1}+wl_{2}}\right\rangle \;, \end{array}$$ (13)
  where the brackets stand for the average of the relative water-use efficiency over the given domain. An agroforestry system that is as water-use efficient as growing the two species in separate monoculture plots will have *ε*_*W*_ = 1. The lower *ε*_*W*_, the more efficient the agroforestry system is in comparison to two separate monoculture plots of the two species.

### Numerical methods

We used two main numerical methods to investigate our model’s Eqs (1)–(4):

**Numerical time integration (also called direct numerical simulation):** Here we choose initial condition for (*B*_1_, *B*_2_, *s*_1_, *s*_2_) and integrate Eqs (1)–(4) numerically using implicit pseudo-spectral time integration [38]. For verification of our results, we use a semi-implicit method, where the spatial discretization is done by the finite element method, which is implemented in pde2path [39] in MATLAB.
**Continuation of steady solution branches:** Here we aim to directly compute steady states by dropping the time dependence and hence solving the algebraic system after discretizing space. We use the software package pde2path for numerical continuation and bifurcation analysis [40–42] to explore the bifurcation diagram of the system by a continuation from the bare-soil solution of our system (*B*_1_ = *B*_2_ = 0, *s*_1_ = *s*_2_ ≠ 0). We choose the precipitation *P* as the continuation parameter and obtain the solution branches over a domain of size Ω = (3.00 × 1.75)*m*, chosen for the following reasons:
  1. Moderately small, as on too large domains the continuation of patterns often runs into numerical problems (for instance, due to many small eigenvalues and an “abundance of patterns”; see, for example, the discussion in ref [43]).
  2. Large enough to accommodate the “basic” patterns (see discussion in the Results section).
  The software **pde2path** uses arclength-continuation, which in particular can deal with saddle-node (fold) bifurcations, where solution branches turn back in parameter space. During the continuation, we compute the eigenvalues of the linearization close to zero and thus obtain the linear stability of the associated solutions.
  Points where eigenvalues cross the imaginary axis yield bifurcation points. Checking the branches that bifurcate from a given bifurcation point, we systematically unfold the set of steady solutions, including their stability properties.

## Results

### Emergence of periodic patterns

Periodic vegetation patterns emerge under conditions of water stress [19]. Fig 3 shows a partial bifurcation diagram that displays the existence and stability ranges of uniform and periodic solutions of Eqs (1)–(4) along the rainfall gradient. At low precipitation rates the only solution that exists describes bare soil (black curve in Fig 3). That solution is stable up to the bifurcation point BP1 (corresponding to *P* ≈ 200*mm*/*y*), where a spatially uniform state of herbaceous species appears in a supercritical bifurcation (red branch). Because the herbaceous species has a significantly higher growth rate, compared to the woody species, it is the first to colonize bare soil as precipitation is increased (compare with the blue solution branch that describes uniform woody vegetation). At higher precipitation values, the uniform herbaceous species branch bifurcates in a subcritical bifurcation (BP2) to an unstable uniform mixed woody–herbaceous branch (magenta). This branch passes through a saddle-node bifurcation at *P* ≈ 280*mm*/*y*, and gains stability in a Turing bifurcation [35, 44, 45] at BP3 (*P* ≈ 700*mm*/*y*), from which three mixed patterned states bifurcate (cyan, green, and turquoise curves). We note that the mixed homogeneous branch almost overlaps the homogeneous woody branch at high precipitation values, indicating the suppression of the herbaceous species by the woody species, mainly by overshading.

We base the naming of the patterned branches on the woody species, and thus refer to the mixed-patterned states that bifurcate at BP3 from the uniform mixed woody–herbaceous branch as stripes (green), gaps (cyan), and spots (turquoise). The mixed patterned states have distinct precipitation ranges of existence and stability. Gaps are stable at high precipitation ranges, stripes cover the intermediate range, and spots are stable at the lower range of precipitation. For the given domain size, we find a precipitation range where the three alternative patterns are stable. The stability ranges of these solutions can in principle decrease on larger domains where more pattern become possible, but in our problem this effect is very small, i.e., our basic domain is large enough to yield robust stability results.

**Fig 4:**
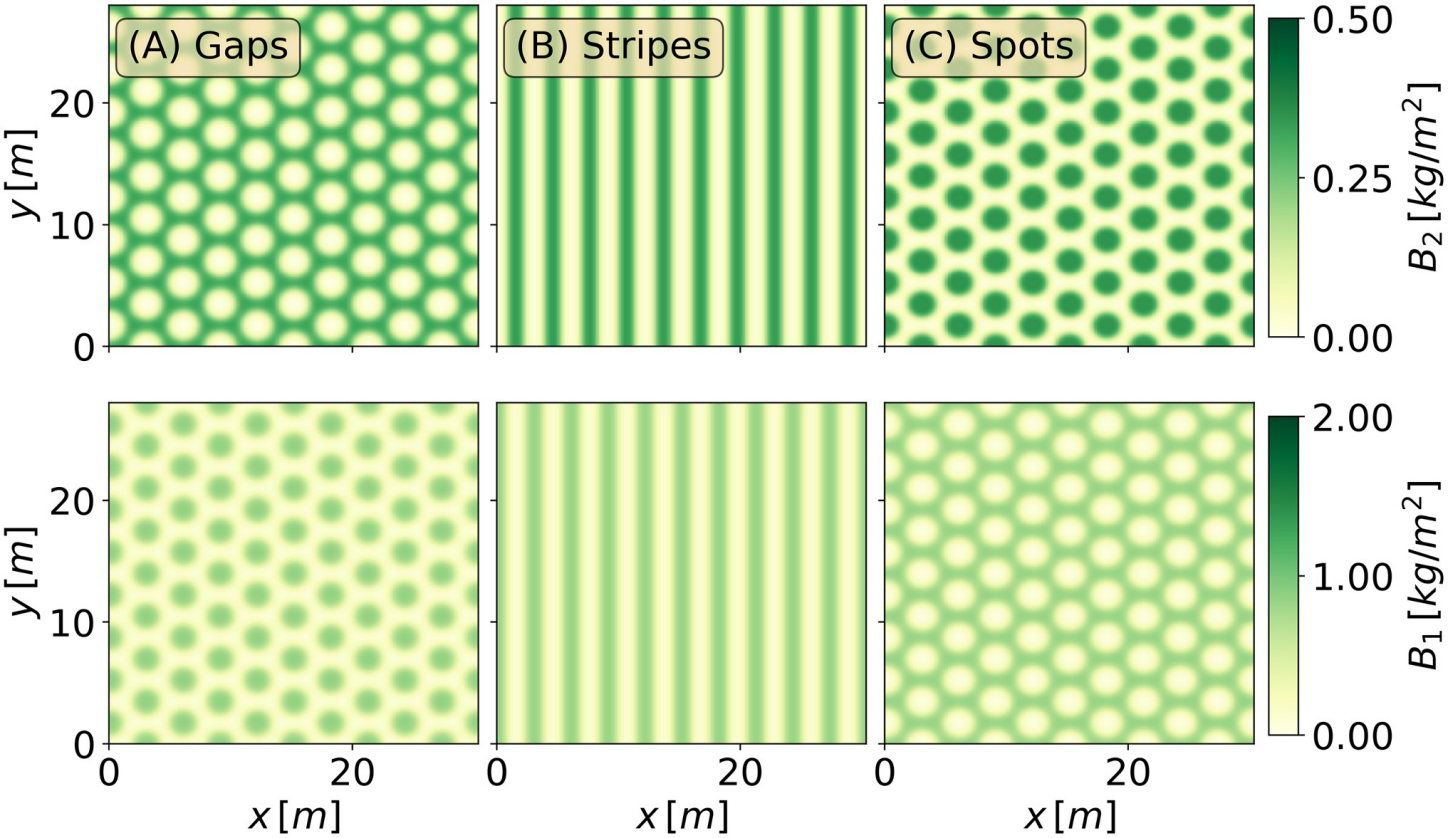
The three alternative patterns at tristability range. Three alternative states for *P* = 600*mm*/*y* over a domain of Ω = (30.0 × 30.0)*m*. The upper row depicts the herbaceous species, and the lower row the woody species.

Fig 4 shows the gaps, stripes and spots at *P* = 600*mm*/*y*, as obtained by integrating the model equations over a larger domain Ω = (30.0×30.0)*m*, starting from initial conditions that correspond to the numerically continued solutions shown in Fig 3.

**Fig 5:**
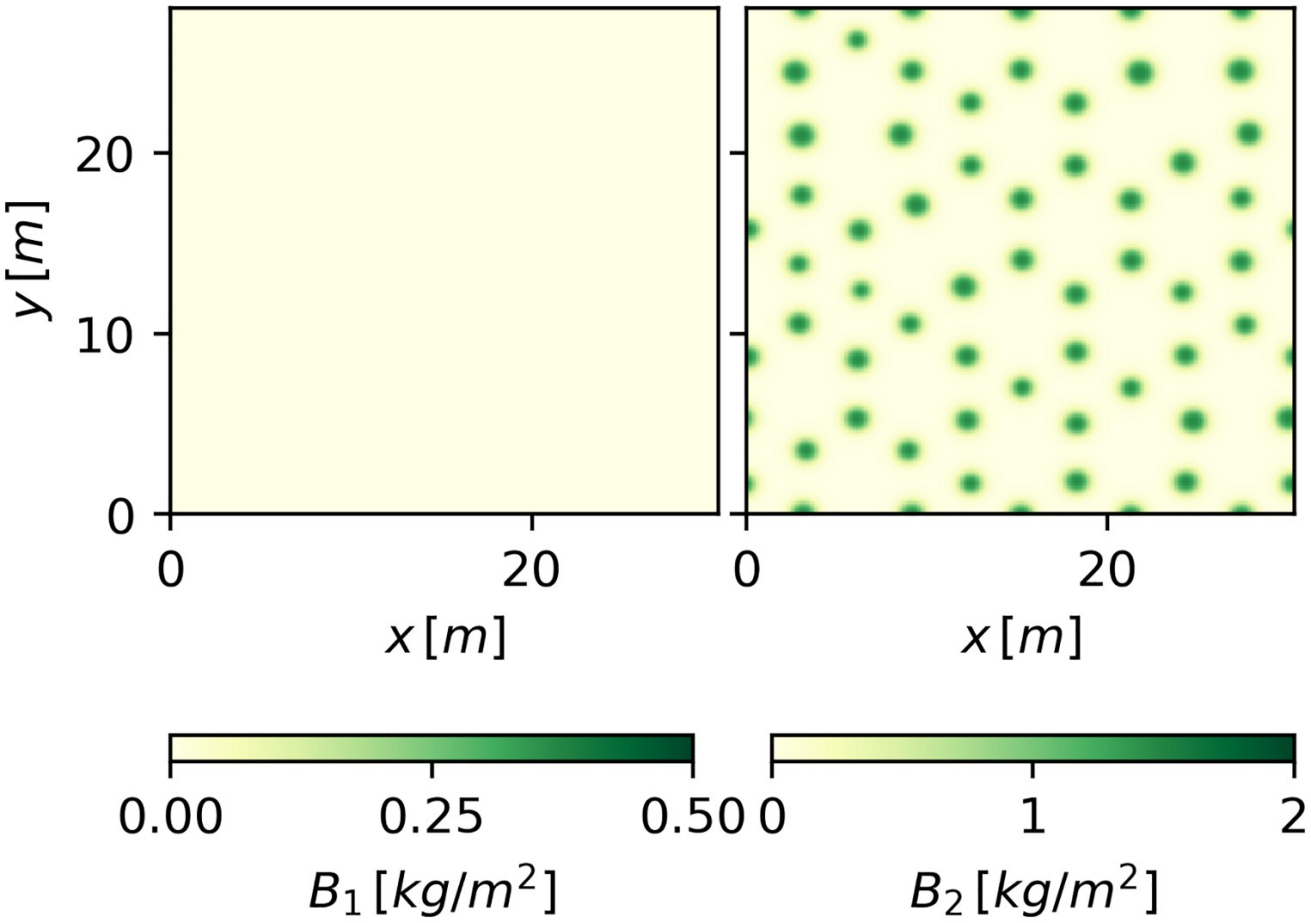
Woody-only spots pattern. Results of direct numerical simulation at *P* = 170*mm*/*y*, starting from an initial conditions near BP4.

At low precipitation values, close to that of BP1 (*P* ≈ 200*mm*/*y*) where the solution branch describing uniform herbaceous vegetation ceases to exist, the herbaceous species no longer survive also in patterned forms; the mixed spot-pattern branch (turquoise) bifurcates at BP4 to an unstable woody-only spot-pattern branch (orange), and an additional mixed spot-pattern branch of longer wavelength (olive) bifurcates at BP5 to a stable longer-wavelength woody-only spot-pattern branch (brown). Fig 5 shows the results of direct time integration of the model equations on a larger domain at *P* = 170*mm*/*y*, indicating an approach to a woody-only spot pattern of longer wavelength, corresponding to the brown branch in Fig 3.

### Performance of different configuration patterns

**Fig 6:**
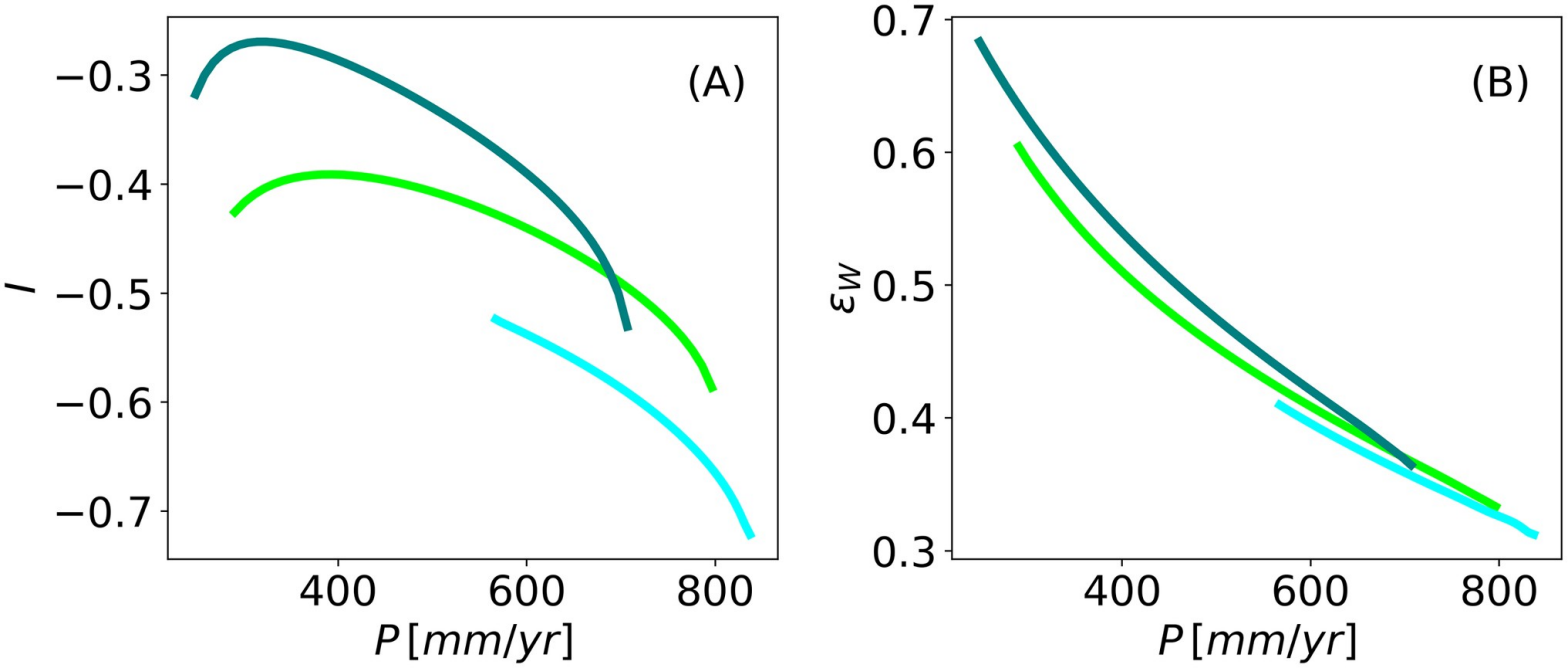
Performance metrics for the model. Performance metrics for the three alternative states, gaps, (light blue curve), stripes (light green curve), and spots (turquoise curve), along with the precipitation range, where only the stable parts of the corresponding solution branches are plotted.

The two indices *I* and *ϵ*_*w*_ that were introduced in the Agroforestry metrics subsection allow us to evaluate the performance of the three patterned states from an agricultural perspective. Fig 6 shows the dependence of these indices on the precipitation parameter for the three states. Although the three alternative states do not differ significantly in their water-use efficiency, as measured by the *ϵ*_*w*_ index, there is a substantial difference in the *I*-index: the spot pattern outperforms the two other patterns, especially at low precipitation values. Put in different words, the woody species in a configuration of a spot pattern is the least competitive with the herbaceous species, thereby allowing a higher crop yield.

**Fig 7:**
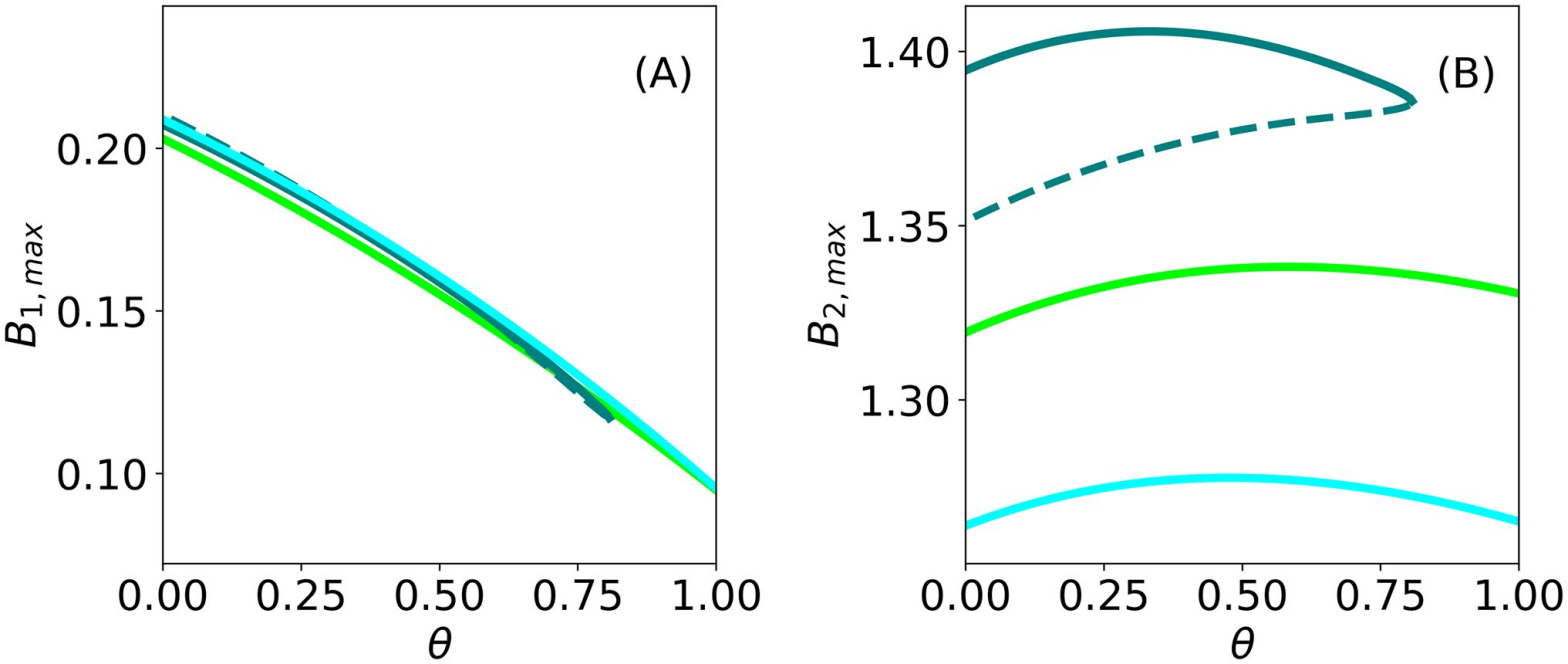
Continuation over the niche-separation parameter. Continuation over the parameter *θ* of the three patterned states, gaps(light blue curve), stripes (light green curve), and spots (turquoise curve), for *P* = 600 *mm*/*y*. Solid (dashed) lines denote stable (unstable) solutions.

The results shown in Fig 6 were obtained with complete niche separation, i.e. with *θ* = 0, where the woody species takes up water from the bottom soil layer only. We then considered the case where the woody species also takes up water from the top layer. Fig 7 shows how the woody and herbaceous species respond to increasing niche overlap, i.e. to water uptake by the woody species from the top soil layer too. Quite expectedly, the increase of niche overlap *θ* has a negative effect on the herbaceous species, reducing its biomass, as Fig 7a shows. More surprising is the fold of the spot solution branch at high niche overlap in Fig 7b, and the persistence of the other two pattern types. While a mechanistic explanation for the fold calls for additional studies, this result shows that, when aiming at a spot configuration, it may be important to minimize niche overlap by judicious choice of the woody plant species.

### Resilience of the patterned states to drier climate

The projections for a warmer and drier climate (REFS) motivate the study of the agroecosystems’ response to gradually decreasing rainfall.

We simulate a developing drier climate over a period of 100 years by solving the model equations with a linearly decreasing precipitation, *P*(*T*) = *P*_0_−*P*_1_ *T*, starting at *P*_0_ = 600*mm*/*y* and decreasing rates of *P*_1_ = 2 and 4*mm*/*y*^2^.

**Fig 8:**
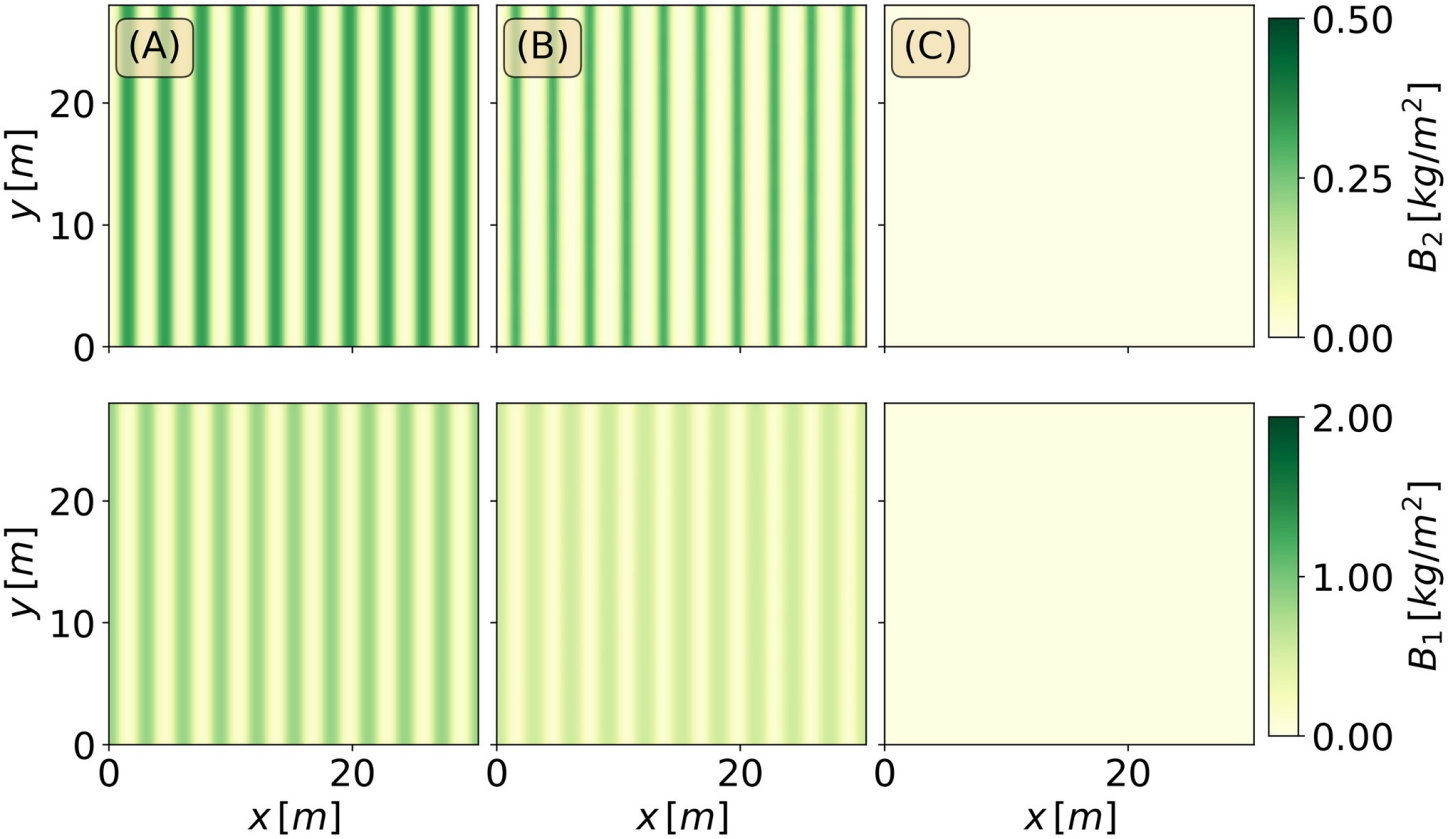
Precipitation downshift for the stripes patterns. Time integration of the stripes patterns for a downshift of the mean annual precipitation from *P* = 600*mm*/*y* (in (A)), to *P* = 400*mm*/*y* (in (B)), and to *P* = 200*mm*/*y* (in (C)), over a period of *T* = 100*y*.

**Fig 9:**
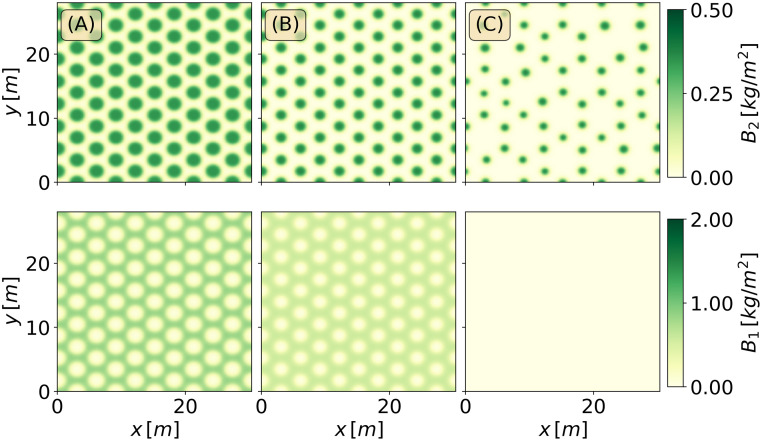
Precipitation downshift for the spots patterns. Time integration of the spots patterns for a downshift of the mean annual precipitation from *P* = 600*mm*/*y* (in (A)), to *P* = 400*mm*/*y* (in (B)), and to *P* = 200*mm*/*y* (in (C)), over a period of *T* = 100*y*.

Fig 8 shows the response of an initial mixed stripe pattern obtained from a numerically continued solution. Decreasing the precipitation at a rate of *P*_1_ = 2*mm*/*y*^2^ for 100 years (down to *P* = 400*mm*/*y*) results in a persistent mixed stripe pattern involving the two species, while a faster decrease, at a rate of 4*mm*/*y*^2^, results in total mortality and agroecosystem collapse. Fig 9) shows the response of an initial mixed spot pattern under the same conditions of precipitation decrease. Like in the case of initial stripe patterns, a moderate decrease to 400*mm*/*y* results in a persistent mixed spot pattern involving the two species. However, a faster decrease to 200*mm*/*y* over a period of 100 years does not result in ecosystem collapse; the herbaceous vegetation dies out but the woody vegetation persists, keeping the resilience of the agroforestry system by preventing further degradation processes, such as soil erosion (not modeled here).

## Discussion

The biomimicry hypothesis suggests that human-made land use systems will benefit if they mimic the resource-use patterns of natural ecosystems adapted to the local environmental conditions [16]. Hence, incorporating knowledge obtained from investigations of natural ecosystems into agroecosystems design may assist in the development of sustainable alternatives to industrialized agricultural systems [31]. Using innovative mathematical models can contribute to our understanding of the complex dynamics manifested in such systems [46].

Agroforestry systems are commonly studied using simulations that take into considerations the whole complexity of the system and parametrize each of the plant processes. In those simulations, herbaceous species respond to the template of the woody species, but self-organization processes that result in woody patterns have not been studied. We presented in this study a new conceptual approach to agroforestry modeling, in which the spatial patterns result from spatial self-organization, rather than being imposed. Accordingly, we allow the system to quickly converge to its long-term stable state, reducing the risk of long and costly transients or even collapse to bare soil.

Mixed woody–herbaceous ecosystems are widespread in semiarid environments. The apparent stable coexistence of two competing species appears to be in contrast to the exclusion principle [47]. However, Walter et al. [48] proposed the two-layer hypothesis to explain the coexistence of woody and herbaceous species. The two-layer hypothesis states that the different rooting depths of the two species establish niche separation between them. This hypothesis is particularly suited for explaining the stability of mixed woody–herbaceous ecosystems in drylands [49]. Indeed, a recent study suggests that below mean annual precipitation of *P* ≈ 650*mm*/*y*, woody–herbaceous ecosystems are stabilized by the water constraints, without the need to account for disturbances such as fire and herbivory [50]. Thus, it is imperative to address the vertical dimension of the soil when treating the coexistence of woody and herbaceous species in xeric environments.

In this paper, we explored how the alternative spatial patterns to which woody–herbaceous ecosystems can converge, gaps, stripes and spots, may influence the bioproductivity, water-use efficiency, and resilience of agroecosystems. We presented here a mathematical model for mixed woody–herbaceous ecosystems that conforms to the two-layer hypothesis. Our main findings are: (1) Spot patterns are advantageous over stripes and gaps in water use-efficiency and especially in producing higher crop yields. (2) Spot patterns are more resilient to drier climates, as woody spot patterns (turquoise, olive and brown solution branches in Fig 3) remain viable even when the herbaceous vegetation dies out, thereby preventing irreversible desertification processes such as soil erosion. (3) Increasing niche overlap, by assuming water uptake by the woody species from the top soil layer too, results in the termination of the spot-pattern solution branch and in transitions to the less favorable stripe and gap configurations. The first two results suggest that setting a dryland agroforestry system in a spot woody pattern is advantageous over the common alley (stripe)-cropping practice, in which crop stripes alternate with stripes of trees, but the third result indicates that for such spot configurations minimizing the niche overlap by careful choice of the woody plant species may be important.

We focused in this model study on the comparison of three different spatial configurations: hexagonal gap patterns, stripe patterns and hexagonal spot patterns. To this end it was sufficient to consider small systems in the numerical-continuation studies (Fig 3), and verify the results with direct model simulations on large systems. However, in large systems, and farther away from the Turing instability, additional stable pattern solutions generally exist that share the same configuration. These solutions, which differ by their wavelength, offer some flexibility in determining the configuration of woody-vegetation to be planted. Close to the Turing instability the parameters that control the patterns’ wavelengths are the soil-water diffusion coefficient *D*_*s*_ and the root to shoot ratio *E*_2_ as both of them affect the soil-water flux (the later affects local water uptake and thus soil-water gradients). As the precipitation rate *P* is further decreased, additional stable solutions with longer wavelengths appear [51–53]. These solutions suggest the possible establishment of sparser agroforestry configurations of woody-vegetation with higher values of the *I* index.

The model given by Eqs (1)–(4) captures a single pattern-forming feedback, associated with soil-water diffusion. This model applies to ecosystems with sandy soil and laterally confined root zones [19]. We expect our results to apply to other forms of water transport, such as overland water flow and water conduction by laterally spread roots, as the pattern-forming feedback associated with these water-transport mechanisms also produce the same sequence of patterns along the rainfall gradients: hexagonal gap, stripe and hexagonal spot patterns [19, 54].

The consideration of additional water-transport mechanisms and the pattern-forming feedbacks associated with them [19] is particularly interesting because of the possible interplay between different feedbacks. Such an interplay may result in woody plants acting as ecosystem engineers and facilitating the growth of herbaceous vegetation under conditions of water stress [25, 55, 56]. We note that taking into account the seasonal and intermittent rainfall conditions may lead to additional differences in the evapotranspiration of the three alternative patterns [32, 33]. Nevertheless, our results suggest that for constant rainfall the patterns in which the woody species are planted influence their competition with the herbaceous species. Furthermore, as each one of the patterns has different existence and stability ranges with respect to the environmental conditions, it can be valuable to recognize the different spatial patterns that are observed in the natural ecosystems. Therefore, we advocate pattern formation theory as a framework for investigating and comparing different templates for agroforestry plantations.
